# Negative Life Events Vary by Neighborhood and Mediate the Relation between Neighborhood Context and Psychological Well-Being

**DOI:** 10.1371/journal.pone.0093539

**Published:** 2014-04-08

**Authors:** Katherine King, Christin Ogle

**Affiliations:** 1 Environmental Public Health Division, Environmental Protection Agency, Chapel Hill, North Carolina, United States of America; 2 Community and Family Medicine, Duke University, Durham, North Carolina, United States of America; 3 Department of Psychology and Neuroscience, Duke University, Durham, North Carolina, United States of America; Hunter College, City University of New York (CUNY), CUNY School of Public Health, United States of America

## Abstract

Researchers have speculated that negative life events are more common in troubled neighborhoods, amplifying adverse effects on health. Using a clustered representative sample of Chicago residents (2001–03; n = 3,105) from the Chicago Community Adult Health Survey, we provide the first documentation that negative life events are highly geographically clustered compared to health outcomes. Associations between neighborhood context and negative life events were also found to vary by event type. We then demonstrate the power of a contextualized approach by testing path models in which life events mediate the relation between neighborhood characteristics and health outcomes, including self-rated health, anxiety, and depression. The indirect paths between neighborhood conditions and health through negative life event exposure are highly significant and large compared to the direct paths from neighborhood conditions to health. Our results indicate that neighborhood conditions can have acute as well as chronic effects on health, and that negative life events are a powerful mechanism by which context may influence health.

## Introduction

Negative life events are powerful potential triggers in producing stress-related health effects [1], [2]. These stressful and potentially traumatic events occur more frequently and earlier among those with low socioeconomic status [2]–[7]. In a review of mechanisms linking psychological factors and health, Miller and colleagues [8] hypothesized that negative life events may also be more common in disadvantaged neighborhoods. Variation in neighborhood conditions due to segregation may also be a key contributor to health disparities [9], [10]. Context may to some extent itself predict or condition stress, emotions, and coping behaviors directly [11]–[13]. We provide the first evidence that neighborhood conditions are related to the prevalence of recent negative life events, and demonstrate the importance of this link for psychological well-being.

Most survey-based research on life events has implicitly treated life event risk as a risk faced by individuals. We argue that individuals are embedded in communities, that some communities experience disproportionate social, demographic, and physical hazards (6–8), and that these hazards are intertwined. The phrase “riskscape” [14] conveys the idea that particular physical and social risks (or resources) in a place are not independent but rather interact and accumulate. This growing appreciation that contextual risk is more than the sum of risks from individual toxicants or stressors has given rise to a new emphasis on documenting the nature of these cumulative exposures [15]. Just as a concentration of poverty, social dysfunction, and crime in a community may lead to worse health outcomes [16], we expect that certain neighborhood conditions may independently increase the risk of future negative life events. Meanwhile, negative life events may also have triggered downward social mobility. Downward social mobility may co-occur with moves to worse neighborhoods or staying in declining neighborhoods. Hardships and traumatic experiences in certain communities may be associated with subsequent social, psychological, and health problems. Thus, we hypothesize strong links between recent negative life events and neighborhood conditions.

Life events are not purely exogenous shocks but rather are correlated with exposures to other risks and resources in one's environment. Variation in recent experience of negative life events is one important mechanism by which deleterious neighborhood effects may relate to downstream health outcomes. However, existing research has not yet assessed whether neighborhood conditions are linked with negative life event experience. Using data from a representative sample of adults clustered in 343 contiguous Chicago neighborhoods, we report the prevalence of a variety of negative life events in a representative urban sample and examine which life events are most clustered geographically. To facilitate comparison with prior literature, we present individual sociodemographic predictors of any recent life event, followed by predictors of 4 categories of life events, based on typologies commonly used in the trauma literature. We report on how a wide range of neighborhood conditions is differentially related to exposure to the life event categories. Finally, we use path models to demonstrate that the indirect association of neighborhood conditions and multifaceted psychological well-being mediated through life event experience are stronger than the direct neighborhood “effects.”

### Life Events in Spatial Context

#### Importance of Life Events

Exposure to negative life events has been associated with a variety of adverse physical and psychological health outcomes, including symptoms of depression [17] and post-traumatic stress disorder (PTSD) [18], reduced life satisfaction [19], inflammation [20], and chronic physical conditions [21]. In addition, research has shown that traumatic experiences over the life course can exert greater adverse effects on psychological and physical health outcomes compared to single incident events [22]–[24]. Given that the majority of individuals exposed to stressful and traumatic events experience more than one event during their lifetime [25], it is important to understand the cumulative effects of exposure to multiple negative life events as well as the differential impact of exposure to various types of events separately.

#### Life Events in Context

Life events have typically been viewed as individual risks. However, life event risks and experiences take place in physical locations which carry different risks for exposure to certain types of events. Negative life events are more common in places that are undergoing periods of violence and hardship, such as war and natural disaster. Dropping out of school and teenage childbearing are also much more common in troubled neighborhoods [26]. Durkheim's classic work on suicide [27] demonstrated differences in suicide rates between Catholic and Protestant places. Also, characteristics of individuals' social networks (embedded in place) may increase the risk of experiencing a negative life event. For instance, older adults are particularly likely to experience the death of social contacts, simply because many of the people they know are also older adults.

Thus, life events occur within socially- and spatially-variant contexts. While a few papers have discussed a specific context in which life event data were collected (*place*; i.e. [28]), research has rarely considered how various conditions in locations in which respondents spend time (*space*) may relate to their experience of life events. Life experiences may vary across neighborhoods as a result of variations in worldviews, economic opportunities, physical safety, social support, and access to resources (e.g., transportation, health care, child care). Certain aspects of family dynamics, such as the role of older adults in the family structure and the amount of responsibility placed on children and young adults, may also vary culturally across neighborhood contexts. These differences may in turn influence the risk (and impact) of negative life events. Only one prior study looked at life events in context, finding neighborhood disadvantage and disorder amplified the association between negative life events and the onset of major depression among African-American women [29].

#### Health in Context

Advancing research concerning contextual effects on health will require greater attention to concrete mechanisms by which specific features of neighborhoods “get under the skin” [9], [30]. One way to begin looking for contextual causal mechanisms is to investigate the geographic clustering of risks, resources, and outcomes. When residential neighbors have more similar values of a variable than those who live farther away (geographic clustering) [31], this suggests the potential for a neighborhood mechanism. To some extent, geographic clustering can be spurious – the result of composition effects. For instance, neighborhoods populated by older adults will have higher mortality because older adults have higher mortality. Neighborhood effects could also be due to selective migration related to the variable being studied. For example, the life event “death of spouse” would be more common in Sunbelt retirement towns because older adults migrate there in later life expecting to live well, not because retirement towns are dangerous. Negative life events may also be contagious, rather than causal or compositional, such as when gang violence results in a string of reprisals. Once geographic clustering has been documented, identifying which of these mechanisms is at play and to what extent should be an important research goal.

**Figure 1:**
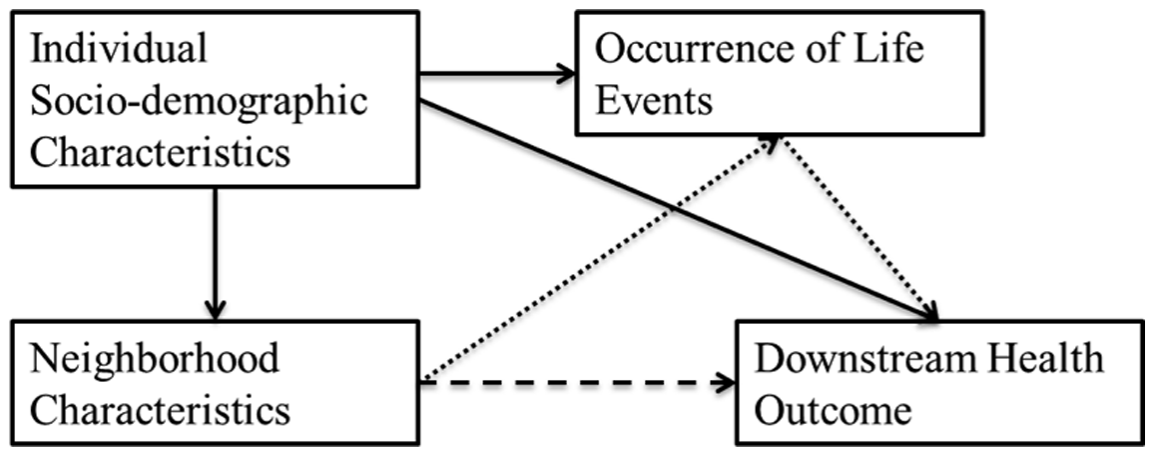
Acute Versus Chronic Effects of Neighborhood Conditions. If the event does not occur, health outcomes may be affected negatively by stress and by the opportunity costs of steps taken to avoid the event or to mitigate its effects if it did occur. For example, fear of crime may lead someone to avoid leaving home to make friends, or to install a burglar alarm. This is the *chronic* path. In contrast, if one is criminally victimized, there is a potential for immediate effects on psychological or physical health. This is the *acute* path.

To illustrate, crime rates vary substantially across intra-urban neighborhoods [32], and living in a high-crime area may influence health in multiple ways. The effort to avoid crime may influence health behaviors – to avoid being victimized, residents may avoid walking for exercise or transportation and spending time outdoors. Continual fear of crime may also result in chronic activation of stress processes, which may lead to physiological dysregulation and later disease [33]. Fear of crime may also influence social behaviors such as avoiding interacting with neighbors, community participation, and moving away, triggering a reciprocal effect such that fear of crime induces conditions which may foster social disorganization [34], which in turn leads to higher crime rates. Although prior research has focused on *chronic* pathways like fear, our goal is to examine the *acute* effects of neighborhood conditions, which are experienced only in the instance in which the feared event occurs (e.g. a resident is a victim of a crime) (Figure 1). This distinction between chronic and acute effects applies to many aspects of place, such as fear of police vs. being unnecessarily frisked, traffic stress vs. having an accident, or anxiety while home alone vs. being robbed. It is likely that the health effects of actually experiencing the feared event (the acute exposure) is much greater than the effect of the fear (the chronic exposure), even though far more people are exposed to the chronic than the acute stressor.

#### Pathways

**Figure 2:**
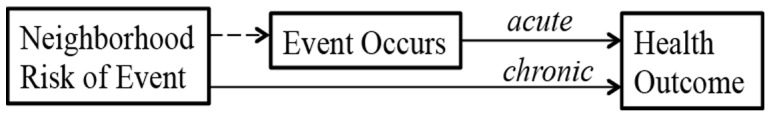
Pathways by which Contextual Life Event Risk May Influence Downstream Outcomes. A: Moderation of neighborhood conditions by occurrence of life event. B: Direct association of neighborhood conditions with outcome. C and D: Indirect association of neighborhood conditions with outcome modified by negative life event risk.

Figure 2 illustrates potential pathways between neighborhood characteristics and health outcomes through life events. Crucially, we do not know the comparative amount that stress- or event-based pathways influence downstream health. As a result, we might overestimate the contextual effect of fear of negative events because sometimes those events actually occur, and both fear of and exposure to the event may influence the same health outcome. Thus life events might mediate and or moderate neighborhood conditions. For example, a higher crime rate increases the risk of victimization, and having been victimized might increase the stress response to exposure to further crime risk. The pathway through life events may also suppress a direct effect. Failing to account for acute as well as chronic effects might thus result in model misspecification. Most neighborhood studies include individual controls and neighborhood predictors, and many recognize potential confounding between the two levels (i.e., between individual and neighborhood income/wealth and race/racial composition [35]). But little attention has been given to potential confounding between individual and contextual stressors. Researchers interested in stress mechanisms typically introduce a neighborhood measure into a model, understanding the direct pathway to be the total effect. Our main goal is to draw attention to a potential additional pathway not previously emphasized in the literature. Increased risk of a negative life event having occurred to respondents in a certain neighborhood (causal or spurious) may result in an individual experiencing the event, and experience of the event may influence their health in an acute way that is distinct from the chronic effect. Alternatively, a negative life event may propel an individual into worse contexts. In other words, life events may mediate/confound/suppress the relationship between neighborhood characteristics and health outcomes. (Confounding and mediation are computationally equivalent in cross-sectional models, and attribution of the effect type depends on the researchers' understanding of the pathways involved [36].)

Specifically, our aim is to illustrate that when neighborhood predictors are entered into a model predicting a health outcome, those neighborhood predictors are correlated with the recent experiences of individuals in prior contexts. For this reason we use retrospective rather than prospective data, and focus on where respondents live at the time of data collection rather than when the event occurred, consistent with the data framework used in research on stress and neighborhood effects on health.

## Materials and Methods

### Sample

To assess the association of negative life events with neighborhood characteristics and health, we use a multilevel probability sample of 3,105 adults age 18 and older, with analyses weighted to represent the Chicago population in 2000. The Chicago Community Adult Health Study (CCAHS) consists of face-to-face interviews (71.8% response rate), systematic social observation, a community survey, and linkage with archival data. The survey elicits individual level data about socioeconomic, psychosocial, behavioral factors, health, and perceived social and physical characteristics of neighborhoods. Neighborhoods in the CCAHS are operationalized as clusters (NCs) of contiguous census tracts, based on the clustered sampling framework of the Project on Human Development in Chicago Neighborhoods [37] and reflecting physical barriers, local cultural knowledge, and cluster analyses of census data so that the NCs are relatively homogeneous and cover the entire city [38]. On average 9 respondents live in each of the 343 NCs. Analyses are weighted to represent Chicago's 2000 Census population in terms of age, race/ethnicity, and sex. The sociodemographic composition of the sample has been reported elsewhere [13]. There are substantial numbers of minorities and a broad range of adult ages and socioeconomic statuses [39]. Data collection for the CCAHS was approved under the University of Michigan Health Sciences and Behavioral Sciences Institutional Review Boards. Respondents gave written informed consent, and data were deidentified prior to public release.

### Measurement

#### Negative Life Events

Respondents were asked about their experience of 16 specific negative life events in the last 5 years (Table 1). CCAHS selected events using a preliminary field study based on the Duke Life Events Inventory [1], [40]. To examine the extent to which neighborhood conditions differentially relate to various types of life events, events were classified into several dichotomous categories (Table 1). First, events were classified as *respondent-directed* or *other-directed* (i.e., events that affected the self primarily through their impact on others). Negative and traumatic events that are directly experienced are generally associated with greater adverse outcomes (e.g., symptoms of depression, PTSD) compared to negative and traumatic events directed at others [41]. Second, in accordance with the A1 PTSD diagnostic criterion (DSM-IV-R, [42]), an event was categorized as a *trauma* if it was life threatening for the individual or someone else involved. Events that meet the A1 criterion are generally associated with greater PTSD symptoms than non-A1 events [43], [44]. Events that were non-life threatening were classified as a *hardship*.

**Table 1:** Categories of Life Events and Prevalence Rates of Recent Negative Life Events Ranked by Level of Neighborhood Similarity.

	Category					ICC	
Event	Directed at Respondent	Directed at Other	Trauma	Hardship	Prevalence (%)	Unadjusted	Sociodemographic
Lost Job, Someone in HH		X		X	2.0	0.181	0.171
Life-Threatening Illness/Accident of Spouse/Child		X	X		0.8	0.132	0.066
Death of Child		X	X		4.8	0.132	0.044
Moved to Worse Neighborhood	X			X	9.8	0.103	0.087
Physical Assault	X		X		3.2	0.098	0.050
Serious Financial Difficulties	X			X	23.0	0.082	0.030
Robbed/Burglarized	X		-	-	12.1	0.080	0.077
Life-Threatening Illness/Accident of Respondent	X		X		5.7	0.077	0.061
Legal Trouble	-	-		X	16.6	0.076	0.061
Unemployed >3 Months	X			X	16.4	0.074	0.032
Unemployed >3 Months, Someone in HH		X		X	11.5	0.059	0.043
Divorced				X	4.8	0.040	0.048
Lost Job	X			X	13.7	0.040	0.033
Life-Threatening Illness/Accident of Someone Else Close		X	X		36.0	0.038	0.026
Death of Someone Close		X	X		4.7	0.025	0.034

* Results were adjusted for socio-demographic variables including race/ethnicity, gender, first generation immigrant status, age, education, and annual income.

CCAHS, 2001–03.

#### Neighborhood Conditions

The 11 measures of neighborhood conditions are standardized across NCs. Based on recent research reporting distinct relations between multiple dimensions of contextual socioeconomic conditions and outcomes, neighborhood disadvantage and affluence are assessed separately [38], [45]. Principal components factor analysis of 2000 Census NC-level measures yielded two factors [46]. The neighborhood disadvantage factor loads negatively on high family incomes, and positively on low family incomes, high levels of poverty, public assistance, unemployment, and vacant housing. The affluence factor loads positively on measures of the proportion of employed civilians ages 16 and over in professional or managerial occupations, the proportion of individuals ages 25 and over who have completed 16 or more years of education, and median home values. Disadvantage and affluence capture distinct but correlated aspects of neighborhood socioeconomic status, much as income and education represent distinct aspects of individual socioeconomic status. Many neighborhoods low in disadvantage are also low in affluence [47]. The traditional family structure scale is composed of percent of families headed by females (reverse-coded), and percent of residents married. The physical disorder measure used here comes from the Systematic Social Observation (SSO) [48], [49] component of the CCAHS, in which trained raters assessed 9 ecological conditions (i.e. graffiti, litter, abandoned cars, broken glass) using standardized criteria.

The perceived measures are aggregated to the neighborhood level from individual responses to the Community Survey section of the CCAHS. Scales are based on the PHDCN [50] and use approximately five questions for each scale. Aggregation of reports from all respondents within the NC minimizes the importance of each respondent's response, so that the respondent's own perceptions of neighborhood will not overly influence results [11]. To aggregate individual item responses to factors at the neighborhood level, empirical Bayesian hierarchical linear models were used [51]. The procedure adjusts for missing items, controls for individual socioeconomic characteristics, and improves neighborhood-level estimates by borrowing information from across locations [51]. A perceived disorder measure was also studied because both measures are widely used; social stigma can amplify perceptions [52]. The “collective efficacy” scale includes 10 items from two subscales and assesses a shared willingness (social cohesion) to take action to enforce collective norms (social control) [53]. The total victimization scale captures experiences of being a victim of crime (i.e. assault, property theft, robbery) in the neighborhood. Anomie assesses the extent to which residents report a disconnection from basic societal rules (e.g. agreeing with “Laws are made to be broken”). Reciprocal exchange focuses on the exchange of favors, advice, material goods, and information which make up a social support network within the community [54].

#### Sociodemographics

Previous research has shown that individual sociodemographic factors predict both the experience of negative life events [2] and the kinds of neighborhoods in which individuals live [55]. We therefore adjust for individual sociodemographics: race/ethnicity (non-Hispanic black, Hispanic, non-Hispanic other, with non-Hispanic White as the reference category), variables indicating whether the respondent is female and is a first generation immigrant, and dummy variables for age (30–39, 40–49, 50–59, 60–69, and 70 years and over, with 18–29 as the reference group), education (12–15 years or 16+ years, with 0–11 years as the reference category), and annual income (of respondent and their spouse if applicable) (less than $5,000, $15,000–$39,999, $40,000 and over, and missing, with $5,000–$15,000 as the reference category). Categories are used for age in order to highlight non-linearity.

#### Health Outcomes

Three health outcomes were selected as commonly studied with respect to both life events and neighborhoods. Descriptions of the depression (CESD) [56], anxiety [57], and self-rated health [58] measures used are given elsewhere.

### Analytic Plan

The analysis was organized in several stages. First, the prevalence of exposure to each life event in the five years prior to the interview is reported, along with the level of neighborhood similarity of exposure to each event. Neighborhood (i.e. NC) similarity is assessed using intraclass correlations (ICCs), where the classes are neighborhoods. ICCs are reported for each life event category, without and with adjustment for sociodemographics. This method partitions variance in multilevel models attributable to individual versus neighborhood levels as a ratio of the between-neighborhood variance to the sum of the between-neighborhood variance and the between-individual, within-neighborhood variance. The ICC for logit models is calculated as τ/(τ+3.29), where τ is the variance of the random intercepts [59]. ICCs can only be compared within a given dataset, and comparison with other findings from the CCAHS are discussed. Particularly given the level of statistical complexity in the measure and the low prevalence of some events, the ICCs should be treated as general indicators of the magnitude of clustering rather than as absolute ranks.

Second, a binary indicator of whether the respondent has experienced a recent life event is aggregated to the neighborhood level using the method previously described, and the results mapped. Third, population-weighted regression models assess associations between individual-level characteristics (e.g. socioeconomic status) and life event categories. Fourth, mixed-effects models are estimated to assess the relationship between neighborhood characteristics and life event categories. Mixed-effects models are necessary because multiple respondents living in each neighborhood violates the regression independence assumption. These population-average robust models with population weights include controls for individual socio-demographics not shown. Fifth, to check for sensitivity to neighborhood spatial boundaries, the association between life events and disadvantage is assessed at various spatial scales. Finally, path analyses of the associations among 2 of the most frequently studied [60] neighborhood characteristics (disadvantage and perceived disorder), any recent life event, and 3 health outcomes (self-rated health, depression, and anxiety) are presented. These population-weighted models were estimated using Mplus software [61], with controls for individual sociodemographics not shown.

## Results

Overall, 72.7% of the sample experienced at least one negative life event in the last 5 years. Table 1 reports the prevalence of each life event in Chicago in 2001–3. Frequencies of individual life events varied widely across event type, with some events occurring among less than 1% of the sample (e.g. life-threatening illness/accident of a spouse/child) and other events occurring among more than 20% of the sample (e.g. financial difficulties, life-threatening illness of someone else close). For each event in Table 1, ICCs are reported with and without adjustment for covariates. Previous reports of geographic clustering using the same dataset (CCAHS) indicate that most measures of neighborhood physical and social conditions have ICCs above 0.13, some negative emotions (e.g. pessimism) have ICCs of 0.06–0.09, while some physical health and psychological measures have ICCs under 0.05 [13], [62]. Thus, the level of clustering of job loss without adjustment for covariates (0.18) is substantial, almost reaching the range of purely neighborhood-based characteristics such as perceived violence in the neighborhood (0.23). The clustering of death of child (0.13) and life-threatening illness/accident to spouse/child (0.13) approximates that of neighborhood-level variables with stronger links to individual characteristics, such as perceived social cohesion (0.14). A moderately high level of clustering was observed for another group of life events, including a move to a worse neighborhood, assault, financial difficulties, robbery/burglary, life-threatening illness/accident of respondent, legal trouble, and unemployment of respondent or household member), which may be consistent with a high degree of clustering of factors at the individual level, and is comparable to the level of geographic clustering previously reported in the same dataset (CCAHS) for negative emotions [13], drinking [63], and exercise [63]. Finally, events including divorce, personal job loss, life-threatening illness/accident of someone else close, and death of someone close, have lower clustering, similar to rates of clustering observed in the CCAHS for measures such as smoking [63], systolic blood pressure, and hopelessness [13].

To assess whether the comparatively high level of clustering observed for negative life events is primarily driven by the tendency of similar individuals and households to live near each, Table 1 also presents ICCs adjusted for sociodemographic variables. In Chicago much of the demographic sorting is due to racial/ethnic and income segregation. Thus, the difference in the levels of clustering between the unadjusted and adjusted ICCs gauges the contribution of segregation and other sorting by neighborhoods to similarity in the experience of negative life events. When adjustment produces larger ICCs, as for divorce and death of someone close, they are interpreted as resulting from disparities running counter to neighborhood selection (e.g. when Whites report greater fear of crime and also live in safer neighborhoods [63]). Overall, our results indicate that clustering of events persists even when adjusted for sociodemographic clustering, suggesting that life events are spatially dependent in their own right.

**Figure 3:**
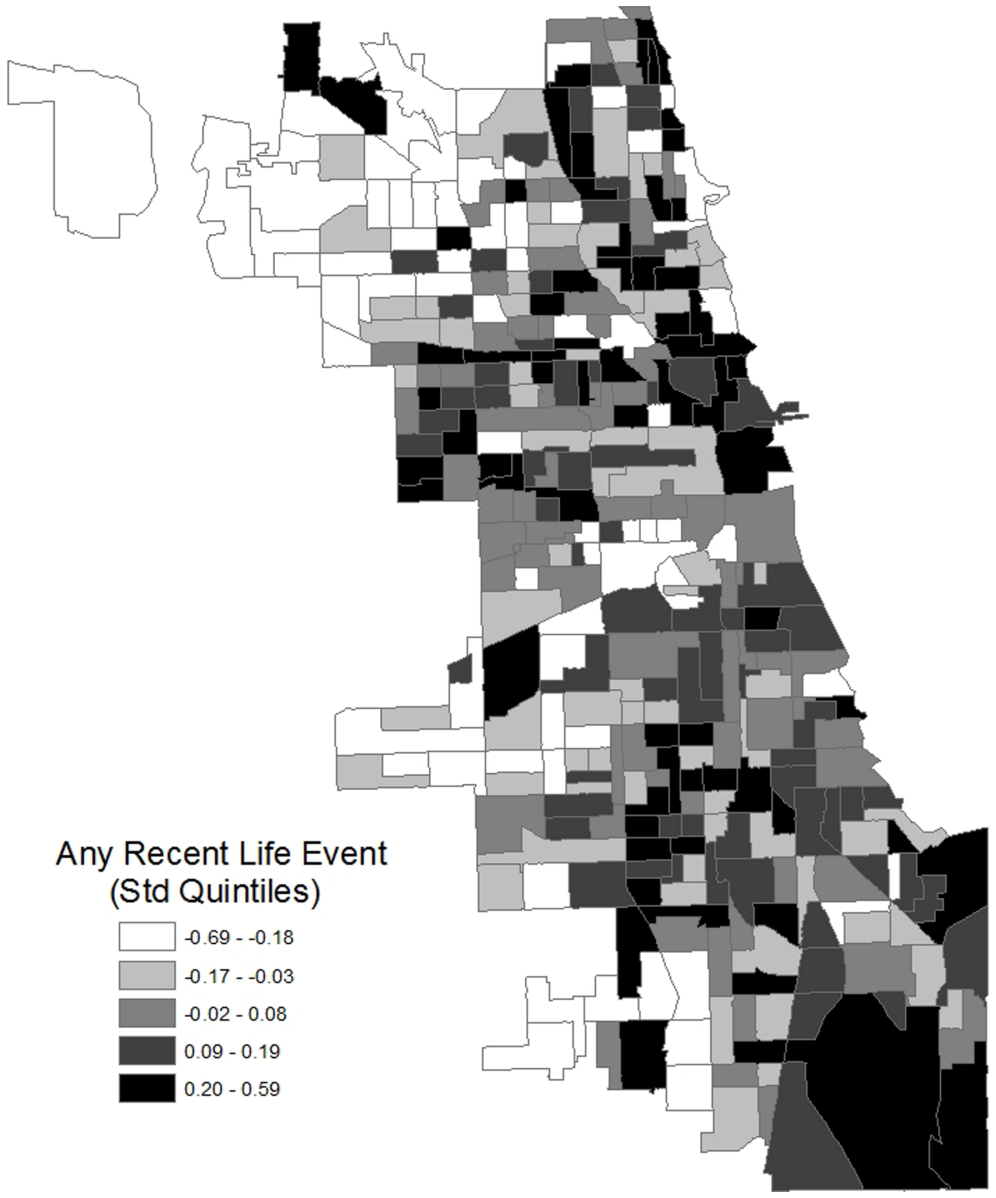
Neighborhood Estimated Experience of a Recent Life Event (Empirical Bayes Estimates; CCAHS, 2001–03).

To visually illustrate the extent to which negative life events are geographically clustered, Figure 3 presents a map of the empirical Bayes estimates of the neighborhood prevalence of any recent life event. The highest quartiles of prevalence are colored darker. Consistent with our other results, life events are not randomly distributed with respect to geography. Rather, a clear spatial distribution is discernible such that recent life events are more common in more disadvantaged areas of the city, as well as downtown. Chicago is bounded by Lake Michigan on the east, and the central business district is centrally located on the shore. Respondents living in the outlying areas of the city (distant from downtown, with more single-family houses and wealthier residents) have experienced few recent life events, adjusted for sociodemographics. These are the same outlying areas previously identified as having high levels of social capital [64]. Meanwhile, the central business district (“The Loop”) area also has a comparatively high prevalence of negative life events, despite the low disadvantage of its residents.

To facilitate comparison with prior research, Table 2 reports associations of socio-demographic variables with life event categories. Consistent with previous research [2], [25], [65], men were significantly more likely to report respondent-directed events, hardships, or any negative life event. Compared to non-Hispanic Whites, non-Hispanic Blacks were more likely to report respondent-directed events and hardships, and Hispanics were more likely to report hardships. Compared to third generation immigrants, first generation immigrants reported far fewer events across all categories, and second generation immigrants reported fewer events overall and respondent-directed events. Older adults also reported having experienced fewer events in the last five years, except for traumas, consistent with findings that negative life events are more common early in the life course [3], [65], [66]. Having moderate ($30,000–$50,000) and higher ($50,000 or higher) income also predicted having recently experienced fewer negative life events, compared to those in the lower income group ($10,000–$30,000), while the very low income group (often the young and the old) was not significantly different from the lower or higher group (not shown).

**Table 2:** Associations of Individual-level Socio-demographic Variables with Negative Life Events (Odds Ratios).

	Directed at Respondent		Directed at Other		Trauma		Hardship		Any Event	
	OR		OR		OR		OR		OR	
Female	0.79	*	0.92		0.86		0.77	**	0.79	*
Race/Ethnicity										
(ref = Non-Hisp White)										
Non-Hisp. Black	1.28	*	0.97		0.84		1.51	***	1.09	
Hispanic	1.32	+	1.04		0.92		1.51	*	1.05	
Non-Hisp. Other	1.33		0.78		0.93		1.01		1.24	
Immigration										
(ref = 3rd+ Generation)										
1st Gen.	0.36	***	0.49	***	0.51	***	0.35	***	0.39	***
2nd Gen.	0.69	*	0.79		0.94		0.69	+	0.70	*
Age (ref = 18–29)										
Age 30–39	0.89		0.99		1.07		0.92		0.97	
Age 40–49	0.77	+	0.99		1.07		0.90		0.85	
Age 50–59	0.65	*	0.97		1.10		0.85		0.83	
Age 60–69	0.28	***	0.73	+	0.85		0.21	***	0.37	***
Age 70+	0.18	***	0.59	**	0.71	+	0.14	***	0.29	***
Education										
(ref = 16+ Years)										
<12 Years	1.22	+	1.10		1.16		1.08		1.17	
12–15 Years	1.01		1.11		1.05		0.94		1.16	
Income										
(ref = $10K-LT$30K)										
LT$10K	1.06		1.02		1.16		1.09		1.02	
$30K-LT$50K	0.67	**	1.04		1.01		0.64	**	0.78	
$50K+	0.62	***	1.00		1.03		0.46	***	0.72	*
Constant	3.07	***	1.26		1.43	+	1.87	***	6.03	***

*** *p<*.001,

** *p<*.01,

* *p<*.05, + *p<*.1 (two-tailed tests); CCAHS, 2001–03.

Given the high levels of clustering reported in Table 2, the next analytic phase focuses on the relation between negative life events and conventionally studied neighborhood measures. Table 3 examines associations of specific features of neighborhoods with life event categories. Several neighborhood conditions (both objective and perceived) are strong predictors of any recent life event. Certain types of events, specifically hardships and respondent-oriented events tended to be related to the neighborhood measures. The types of events which were significantly associated with neighborhood predictors also tended to be those which were more geographically correlated. Respondents do not necessarily live in the same neighborhoods as those emotionally close to them, consistent with there being few significant associations of neighborhood characteristics and events to others. Also, many of the traumatic events are related to illness, which is among the more spatially diffuse phenomena studied here. Residents of disadvantaged neighborhoods have greater odds of having recently experienced any event (except other-oriented events). However, affluence is not associated with the risk of negative life events, consistent with a literature showing distinctive findings for disadvantage and affluence.

**Table 3:** Associations of Neighborhood Conditions with Any Life Event in Category, Neighborhood Clusters**.**

	Directed at Respondent			Directed at Other			Trauma			Hardship			All Recent Life Events		
	Coef.	SE	*p*	Coef.	SE	*p*	Coef.	SE	*p*	Coef.	SE	*p*	Coef.	SE	*p*
**Objective Measures**															
Disadvantage	1.193	0.035	***	1.054	0.030	+	1.062	0.028	*	1.176	0.041	***	1.191	0.037	***
Affluence	0.980	0.023		0.983	0.019		0.988	0.020		0.970	0.024		0.986	0.023	
Nuclear Family Structure	0.853	0.029	***	0.937	0.028	*	0.947	0.026	*	0.855	0.031	***	0.847	0.029	***
% 65 or Older	0.915	0.021	***	1.021	0.018		1.002	0.018		0.944	0.024	*	0.936	0.025	*
Robbery Rate	1.171	0.033	***	1.013	0.025		1.039	0.023	+	1.143	0.035	***	1.160	0.033	***
Disorder	1.148	0.029	***	1.022	0.021		1.043	0.022	+	1.130	0.029	***	1.132	0.029	***
**Perceived Measures**															
Disorder	1.181	0.033	***	1.041	0.023	+	1.055	0.025	*	1.150	0.036	***	1.161	0.033	***
Total Victimization	1.096	0.024	***	1.033	0.019	+	1.049	0.020	*	1.053	0.025	*	1.103	0.025	***
Anomie	1.062	0.025	**	0.975	0.018		0.996	0.018		1.039	0.027		1.045	0.025	+
Collective Efficacy	0.909	0.024	***	1.002	0.019		0.982	0.020		0.925	0.026	**	0.920	0.023	**
Reciprocal Exchange	0.974	0.027		1.013	0.020		0.998	0.019		0.972	0.026		0.986	0.027	
Unadjusted ICC	0.040			0.002			0.012			0.041			0.036		
ICC Adjusted for Sociodemographics	0.016			0.000			0.000			0.013			0.017		

Neighborhood predictors were standardized. Controls for sociodemographics are not shown. Lower ICCs indicate a greater proportion of neighborhood-level variance explained by the predictors.

*** *p<*.001,

** *p<*.01,

* *p<*.05, + *p<*.1 (two-tailed tests).

CCAHS, 2001–03.

Results from separate regressions for each neighborhood-level variable.

Other social composition measures also predict life events: having a greater proportion of older adults in the neighborhood is associated with lower odds of respondent-oriented events, hardships, and overall events. Perhaps neighborhood family composition is related to the likelihood of reporting an event to an acquaintance if non-traditional family composition increases the number of people viewed as family. This finding is also consistent with a protective effect of traditional family contexts. Conditions linked with crime (robbery rate and total victimization) and disorder (objective or perceived) strongly predict greater odds of an event directed at the respondent, hardships, and any event, with weaker or no relationships with events directed at others and traumatic events (probably because of the importance of health in this category). The three measures of neighborhood norms and behaviors show divergent patterns of prediction. Anomie increases odds of events directed at the respondent, but not hardships. Collective efficacy decreases odds of respondent-oriented events, hardships, and any event, while reciprocal exchanges between neighbors are non-significant, lending support to the literature on the benefits of collective efficacy beyond other forms of social interaction.

Several studies have found differences in neighborhood “effects” as a function of how neighborhoods are defined [67]. As a cross-check, additional models (not shown) were estimated in which disadvantage and affluence were assessed in concentric rings (“buffers”) around the respondents' geocoded home addresses with diameters of 250 m, 500 m, 1 km, 1.5 km, 2 km, and 3 km. At each spatial scale, disadvantage coefficients in Poisson models predicting the count of recent life events, adjusting for sociodemographics, were nearly identical (1.1<β<1.2; *p*<.001). Affluence remained non-significant across spatial scale in the buffer models. This indicates that neighborhood definition does not drive results.

Finally, illustrative results from path analyses of neighborhood characteristics (disadvantage and perceived disorder), life events (any recent event), and 3 health measures (worse self-rated health, depression, anxiety) are given in Table 4. In each model, the neighborhood characteristic strongly predicts a recent life event, and a recent life event strongly predicts the health outcome. By contrast, direct associations of the neighborhood variable with health are smaller. Analogous results appeared in models on the subsample of respondents who had lived in their neighborhoods for 5 years or more (not shown). The highly significant Sobel [68] test statistics (which tests the indirect effect of neighborhood conditions on health outcomes through recent negative life events) indicate that recent life events partially mediates the associations of neighborhood conditions and health outcomes.

**Table 4:** Path Analyses of Neighborhood Characteristics, Life Events, and Health Outcomes, Adjusted for Socio-demographics, CCAHS, 2001–03.

			Neigh->LE		Neigh->Health		LE->Health		Sobel Test	
Neighborhood Predictor	Life Event Mediator	Health Outcome	Coeff.	*p*	Coeff.	*p*	Coeff.	*p*	Coeff.	*p*
Disadvantage	Any Recent	Self-Rated Health (Worse)	0.281	***	0.050	+	0.182	***	2.856	***
Disadvantage	Any Recent	Depression	0.280	***	0.037	*	0.218	***	3.474	***
Disadvantage	Any Recent	Anxiety	0.282	***	0.040	*	0.155	***	3.180	***
Perc. Disorder	Any Recent	Self-Rated Health (Worse)	0.260	***	0.090	*	0.354	***	3.175	***
Perc. Disorder	Any Recent	Depression	0.262	***	0.031	*	0.216	***	4.374	***
Perc. Disorder	Any Recent	Anxiety	0.261	***	0.037	**	0.152	***	3.766	***

Neigh = Neighborhood predictor. LE = Life event.

*** *p<*.001,

** *p<*.01,

* *p<*.05, + *p<*.1 (two-tailed tests).

CCAHS, 2001–03.

## Discussion

These findings illustrate the importance of considering acute rather than only chronic pathways between context and health. Experience of negative life events – to varying extents – are patterned by residential neighborhood context. Previous research on the prevalence of stressful and traumatic life events has tended to consider individuals as the unit of analysis. The evidence in this paper suggests a new direction linking research in a variety of fields (criminology, social networks, urban geography) with the literature on neighborhood health and health disparities. Exposure to actual negative life events is a distinct neighborhood-health pathway beyond fear of events, and may serve as an important mechanism by which context influences stress levels.

Several features of the analysis limit the ability to draw causal links between neighborhoods and events, which is *not* our goal. Cross-sectional models cannot directly assess risk of event occurrence in neighborhoods. The survey questions do not specify where the events took place. Future data collection may be able to more explicitly capture how events relate to dynamic spatial and social contexts. Respondents who experience negative events closely linked to place, such as muggings, may be more likely to move to places where they feel safer or where they have no unpleasant memories. Or, negative life events may lead to downward socioeconomic mobility, resulting in residence in less desirable areas. These issues do not detract from our aim to demonstrate the association between recent exposure to negative life events and contextual conditions. Future prospective studies could help establish any dynamic longitudinal relationship between residential attainment/choice and life events.

It is also important to note that respondents are not equally exposed to all events, and not all potential negative events are measured. For instance, divorced respondents must have first been married, and context is associated with both divorce and marriage. Contextual conditions are associated with not only occurrence of an event conditional on eligibility but also eligibility itself. This is because we are interested in the population-level relationship between neighborhood conditions and occurrence of an event. While the structured interview questions covered a broad range of events, many other stressful and potentially traumatic life events, as well as a range of positive events, may be consequential. However, when prompted with an open-ended question, respondents reported relatively few events not previously queried.

Our goal is to quantitatively demonstrate the potential for embedded and interactive experience of conventionally-measured negative life events in risky and resourceful places. Extensive prior ethnographic work has previously described the cumulative nature of life experience burden in poor communities [69]. Whereas ethnographic work can help elucidate stress pathways in individuals and specific communities, spatial demographic work can provide prevalences necessary for comparison across space. The ability to consider spatial variation in life events is required in order to investigate how geographic factors cause life events, how spatial clustering of life events may result in biased estimation of spatial effects, and how life events may lead to further events for those nearby. The results also show that some life events are spatially clustered in ways not driven by poverty, and future work is needed to understand the reason.

Both chronic strain and acute life events may influence physical and mental health over the life course. Preventive efforts may benefit from a careful discourse distinguishing between the impact of fear of events and objective experience of events on health. The work presented here lays a foundation of basic findings on which to bridge research concerning trauma with the literature on neighborhoods and health. We urge stress researchers to consider the embedded nature of specific events within spatial and social network contexts.

## References

[1] Blazer DG, Hughes D, George LK (1987) Stressful Life Events and the Onset on a Generalized Anxiety Syndrome. American Journal of Psychiatry 144: 1178–1183. 3631314 10.1176/ajp.144.9.1178

[2] Lantz PM, House JS, Mero RP, Williams DR (2005) Stress, Life Events, and Socioeconomic Disparities in Health: Results from the Americans' Changing Lives Study. Journal of Health and Social Behavior 46: 274–288. 16259149 10.1177/002214650504600305

[3] Hatch SL, Dohrenwend BP (2007) Distribution of Traumatic and Other Stressful Life Events by Race/ethnicity, Gender, SES and Age: A Review of the Research. American Journal of Community Psychology 40: 313–332. 17906927 10.1007/s10464-007-9134-z

[4] Stansfeld SA, Head J, Marmot MG (1998) Explaining Social Class Differences in Depression and Well-being. Social Psychiatry and Psychiatric Epidemiology 33: 1–9. 9448438 10.1007/s001270050014

[5] Gallo LC, Matthews KA (2003) Understanding the Association Between Socioeconomic Status and Physical Health: Do Negative Emotions Play a Role? Psychological bulletin 129: 10–51. 12555793 10.1037/0033-2909.129.1.10

[6] Fremont AM, Bird CE (2000) Social and Psychological Factors, Physiological Processes, and Physical Health. Handbook of Medical Sociology. New Jersey: Prentice-Hall, Inc.

[7] Pearlin LI, Schooler C (1978) The Structure of Coping. Journal of Health and Social Behavior 19: 2–21. 649936

[8] Miller G, Chen E, Cole SW (2009) Health Psychology: Developing Biologically Plausible Models Linking the Social World and Physical Health. Annual review of psychology 60: 501–524. 10.1146/annurev.psych.60.110707.163551 19035829

[9] Diez Roux AV, Mair C (2010) Neighborhoods and Health. Annals of the New York Academy of Sciences 1186: 125–145. 20201871 10.1111/j.1749-6632.2009.05333.x

[10] King KE, Morenoff JD, House JS (2011) Cumulative Biological Risk Factors: Neighborhood Socioeconomic Characteristics and Race/Ethnic Disparities Psychosomatic medicine. 73: 572–579. 10.1097/PSY.0b013e318227b062 PMC3216672 21862824

[11] Mair C, Diez Roux AV, Galea S (2008) Are Neighbourhood Characteristics Associated with Depressive Symptoms? A Review of Evidence. Journal of epidemiology and community health 62: 940–946. 18775943 10.1136/jech.2007.066605

[12] Taylor SE, Lerner JS, Sage RM, Lehman BJ, Seeman TE (2004) Early Environment, Emotions, Responses to Stress, and Health. Journal of Personality 72: 1365–1393. 15509286 10.1111/j.1467-6494.2004.00300.x

[13] King KE (2012) Aggravating Conditions: Cynical Hostility and Neighborhood Ambient Stressors. Social Science and Medicine 75: 2258–2266. 22995667 10.1016/j.socscimed.2012.08.027 PMC3502671

[14] Morello-Frosch R, Lopez R (2006) The Riskscape and the Colorline: Examining the Role of Segregation in Environmental Health Disparities. Environmental research 102: 181–196. 16828737 10.1016/j.envres.2006.05.007

[15] EPA US (2003) Framework for Cumulative Risk Assessment. Washington, DC: U.S. Environmental Protection Agency, Office of Research and Development.

[16] Pickett KE, Pearl M (2001) Multilevel analyses of neighbourhood socioeconomic context and health outcomes: a critical review. Journal of epidemiology and community health 55: 111–122. 11154250 10.1136/jech.55.2.111 PMC1731829

[17] Kessler RC, Davis CG, Kendler KS (1997) Childhood Adversity and Adult Psychiatric Disorder in the US National Comorbidity Survey. Psychological medicine 27: 1101–1119. 9300515 10.1017/s0033291797005588

[18] Ogle CM, Rubin DC, Berntsen D, Siegler IC (2013) The frequency and impact of exposure to potentially traumatic events over the life course. Clinical psychological science 1: 426–434. 24660131 10.1177/2167702613485076 PMC3958943

[19] Krause N (2004) Lifetime Trauma, Emotional Support, and Life Satisfaction among Older Adults. The Gerontologist 44: 615–623. 15498837 10.1093/geront/44.5.615

[20] O'Donovan A, Neylan T, Metzler T, Cohen B (2012) Lifetime Exposure to Traumatic Psychological Stress is Associated with Elevated Inflammation in the Heart and Soul Study. Brain, behavior, and immunity 26: 642–649. 10.1016/j.bbi.2012.02.003 PMC3322304 22366689

[21] Krause N, Shaw BA, Cairney J (2004) A Descriptive Epidemiology of Lifetime Trauma and the Physical Health Status of Older Adults. Psychology and aging 19: 637–648. 15584789 10.1037/0882-7974.19.4.637

[22] Schnurr PP, Spiro A, Vielhauer MJ, Findler MN, Hamblen JL (2002) Trauma in the Lives of Older Men: Findings from the Normative Aging Study. Journal of Clinical Geropsychology 8: 175–187.

[23] Turner RJ, Lloyd DA (1995) Lifetime Traumas and Mental Health: The Significance of Cumulative Adversity. Journal of Health and Social Behavior 36: 360–376. 8719054

[24] Ogle CM, Rubin DC, Siegler IC (in press) Cumulative exposure to traumatic events in older adults. Aging and Mental Health 10.1080/13607863.2013.832730 PMC3944195 24011223

[25] Breslau N, Kessler RC, Chilcoat HD, Schultz LR, Davis GC, et al (1998) Trauma and Posttraumatic Stress Disorder in the Community. Archives of general psychiatry 55: 913–917. 9672053 10.1001/archpsyc.55.7.626

[26] Crane J (1991) The Epidemic Theory of Ghettos and Neighborhood Effects on Dropping Out and Teenage Childbearing. American Journal of Sociology 96: 1226–1259.

[27] Durkheim E (1997 [1897]) Suicide: A Study in Sociology. The Free Press.

[28] Norris FH, Kaniasty K, Conrad ML, Inman GL, Murphy AD (2002) A Comparison of the Effects of Age on PTSD after Disasters in the United States, Mexico, and Poland. Journal of Clinical Geropsychology 8: 153–173.

[29] Cutrona CE, Russell DW, Hessling RM, Brown PA, Murray V (2000) Direct and Moderating Effects of Community Context on the Psychological Well-being of African American Women. Journal of personality and social psychology 79: 1088–1101. 11138756 10.1037//0022-3514.79.6.1088 PMC1913215

[30] Taylor SE, Repetti RL (1997) Health Psychology: What is an Unhealthy Environment and How Does It Get Under the Skin? Annual review of psychology 48: 411–447. 10.1146/annurev.psych.48.1.411 9046565

[31] Tobler W (1970) A Computer Movie Simulating Urban Growth in the Detroit Region. Economic Geography 46: 234–240.

[32] Perkins DD, Meeks JW, Taylor RB (1992) The physical environment of street blocks and resident perceptions of crime and disorder: Implications for theory and measurement. Journal of Environmental Psychology 12: 21–34.

[33] McEwen BS (1998) Stress, Adaptation, and Disease: Allostasis and Allostatic Load. Annals of the New York Academy of Sciences 840: 33–44. 9629234 10.1111/j.1749-6632.1998.tb09546.x

[34] Woldoff RA (2006) Emphasizing Fear of Crime in Models of Neighborhood Social Disorganization. Crime Prevention and Community Safety 8: 228–247.

[35] Oakes JM (2006) Commentary: Advancing Neighbourhood-Effects Research–Selection, Inferential Support, and Structural Confounding. International Journal of Epidemiology 35: 643–647. 16556642 10.1093/ije/dyl054

[36] MacKinnon D, Krull J, Lockwood C (2000) Equivalence of the Mediation, Confounding and Suppression Effect. Prevention Science 1: 173–181. 11523746 10.1023/a:1026595011371 PMC2819361

[37] PHDCN Project on Human Development in Chicago Neighborhoods. Available: http://www.icpsr.umich.edu/icpsrweb/PHDCN/about.jsp.

[38] Sampson RJ, Morenoff JD, Earls F (1999) Beyond Social Capital: Spatial Dynamics of Collective Efficacy for Children. American Sociological Review 64: 633–660.

[39] King KE (2013) Jane Jacobs and ‘The Need for Aged Buildings’: Neighborhood Historical Development Pace and Community Social Relations. Urban Studies 50: 2407–2424. 10.1177/0042098013477698 PMC3808089 24163485

[40] Hughes DC, Blazer DG, George LK (1988) Age Differences in Life Events: A Multivariate Controlled Analysis. International Journal of Aging and Human Development 27: 207–220. 3243654 10.2190/F9RP-8V9D-CGH7-2F0N

[41] Shmotkin D, Litwin H (2009) Cumulative Adversity and Depressive Symptoms among Older Adults in Israel: The Differential Roles of Self-oriented Versus Other-oriented Events of Potential Trauma. Social Psychiatry and Psychiatric Epidemiology 44: 989–997. 19288036 10.1007/s00127-009-0020-x PMC3547167

[42] Association AP (2000) Diagnostic and Statistical Manual of Mental Disorders. Washington, DC: American Psychiatric Association. Text Revision

[43] Kilpatrick DG, Resnick HS, Freedy JR, Pelcovitz D, et al.. (1998) Posttraumatic Stress Disorder Field Trial: Evaluation of the PTSD Construct-Criteria A through E. In: T. Widiger, A. Frances, H. Pincus, R. Ross, M. First, W. Davis and M. Kline, editors. DSM-IV Sourcebook. Washington, DC: American Psychiatric Press. pp. 803–844.

[44] Ogle CM, Rubin DC, Siegler IC (2013) The impact of the developmental timing of trauma exposure on PTSD symptoms and psychosocial functioning among older adults. Developmental Psychology 49: 2191–2200. 23458662 10.1037/a0031985 PMC3806884

[45] Finch BK, Do DP, Heron M, Bird CE, Seeman TE, et al (2010) Neighborhood Effects on Health Concentrated Advantage and Disadvantage. Health & place 16: 1058–1060. 20627796 10.1016/j.healthplace.2010.05.009 PMC2918664

[46] Morenoff JD, House JS, Hansen BB, Williams D, Kaplan G, et al (2008) Understanding Social Disparities in Hypertension Prevalence, Awareness, Treatment, and Control: The Role of Neighborhood Context. Social Science & Medicine 65: 1853–1866. 10.1016/j.socscimed.2007.05.038 PMC2705439 17640788

[47] King K, Morenoff J, House J (2011) Neighborhood Socioeconomic Characteristics and Social Disparities in Cumulative Biological Risk Factors. Psychosomatic medicine 73: 572–579. 21862824 10.1097/PSY.0b013e318227b062 PMC3216672

[48] Sampson RJ, Raudenbush SW (1999) Systematic Social Observation of Public Spaces: A New Look at Disorder in Urban Neighborhoods. American Journal of Sociology 105: 603–651.

[49] Mair C, Diez Roux AV, Morenoff JD (2010) Neighborhood Stressors and Social Support as Predictors of Depressive Symptoms in the Chicago Community Adult Health Study. Health and Place 16. 10.1016/j.healthplace.2010.04.006 PMC2918682 20434941

[50] Earls FJ, Brooks-Gunn J, Raudenbush SW, Sampson RJ (2007) Project on Human Development in Chicago Neighborhoods: Community Survey, 1994–1995. Inter-university Consortium for Political and Social Research (ICPSR) [distributor].

[51] Raudenbush SW, Bryk AS (2002) Hierarchical Linear Models: Applications and Data Analysis Methods. Thousand Oaks: Sage Publications.

[52] Sampson RJ, Raudenbush SW (2004) Seeing Disorder: Neighborhood Stigma and the Social Construction of “Broken Windows”. Social Psychology Quarterly 67: 319–342.

[53] Sampson RJ, Raudenbush SW, Earls F (1997) Neighborhoods and Violent Crime: A Multilevel Study of Collective Efficacy. Science 277: 918–924. 9252316 10.1126/science.277.5328.918

[54] Portes A (1999) Social Capital: Its Origins and Applications in Modern Sociology. Annual Review of Sociology 24: 1–24.

[55] Massey D (1981) Social Class and Ethnic Segregation. American Sociological Review 46: 641–650.

[56] Hunte H, King K, Hicken M, Lee H, Lewis T (2013) Interpersonal discrimination and depressive symptomatology: examination of several personality-related characteristics as potential confounders in a racial/ethnic heterogeneous adult sample. BMC public health 13: 1084. 24256578 10.1186/1471-2458-13-1084 PMC3845526

[57] Sternthal MJ, Williams DR, Musick MA, Buck AC (2010) Depression, Anxiety, and Religious Life: A Search for Mediators. Journal of Heatlh and Social Behavior 51: 343–359. 10.1177/0022146510378237 20943594

[58] Viruell-Fuentes EA, Morenoff JD, Williams DR, House JS (2010) Language of Interview, Self-Rated Health, and the Other Latino Health Puzzles. American Journal of Public Health e1–e8. 10.2105/AJPH.2009.175455 PMC3110226 21164101

[59] Skrondal A, Rabe-Hesketh S (2004) Generalized Latent Variable Modeling: Multilevel, Longitudinal, and Structural Equation Models. CRC Press.

[60] Entwisle B (2007) Putting People Into Place. Demography 44: 687–703. 18232206 10.1353/dem.2007.0045

[61] Muthén LK, Muthén BO (1998–2011) Mplus User's Guide. Los Angeles, CA: Muthén & Muthén.

[62] King K (2013) Neighborhood Walkable Urban Form and C-Reactive Protein. Preventive Medicine 57: 850–854. 24096140 10.1016/j.ypmed.2013.09.019 PMC3898708

[63] Morenoff JD, Roux AVD, Hansen BB, Osypuk TL (2008) Residential Environments and Obesity: How Can Observational Studies Inform Policy Interventions? In: R. F. Schoeni, J. S. House, G. A. Kaplan and H. Pollock, editors. Making Americans Healthier: Social and Economic Policy as Health Policy. New York: Russell Sage Foundation. pp. 309–343.

[64] Sampson RJ, Morenoff JD, Earls F (1999) Beyond Social Capital: Spatial Dynamics of Collective Efficacy for Children. American Sociological Review 64: 633–660.

[65] Norris FH (1992) Epidemiology of trauma: Frequency and impact of different potentially traumatic events on different demographic groups. Journal of Consulting and Clinical Psychology 60: 409–418. 1619095 10.1037//0022-006x.60.3.409

[66] Terracciano A, McCrae RR, Brant LJ, Costa PT (2005) Hierarchical Linear Modeling Analyses of the NEO-PI–R Scales in the Baltimore Longitudinal Study of Aging. Psychology and Aging 20: 493–506. 16248708 10.1037/0882-7974.20.3.493 PMC2808690

[67] Moudon AV, Lee C, Cheadle AD, Garvin C, Johnson D, et al (2006) Operational Definitions of Walkable Neighborhood: Theoretical and Empirical Insights. Journal of Physical Activity and Health 3: S99–S117. 28834523 10.1123/jpah.3.s1.s99

[68] Sobel ME (1982) Asymptotic Confidence Intervals for Indirect Effects in Structural Equation Models. Sociological Methodology 13: 290–312.

[69] Burton LM, Whitfield KE (2006) Health, Aging, and America's Poor: Ethnographic Insights on Family Co-morbidity and Cumulative Disadvantage. In: J. Baars, D. Dannefer, C. Phillipson and A. Walker, editors. Aging, Globalization and Inequality: The New Critical Gerontology. New York: Baywood.

